# Effects of flushing of dental waterlines in portable dental units on water quality management

**DOI:** 10.1016/j.jds.2024.10.014

**Published:** 2024-10-26

**Authors:** Kunihiro Fushimi, Jun Watanabe, Masahiro Yamada, Jumpei Washio, Nobuhiro Takahashi, Hiroshi Egusa

**Affiliations:** a Division of Molecular and Regenerative Prosthodontics, Tohoku University Graduate School of Dentistry, Sendai, Japan; b Division of Oral Ecology and Biochemistry, Tohoku University Graduate School of Dentistry, Sendai, Japan

**Keywords:** Dental unit waterlines, Flushing, Heterotrophic bacteria, Portable dental unit, Residual chlorine

## Abstract

**Background/purpose:**

Daily flushing of dental unit waterlines is important for infection control. However, the effect of flushing on water quality management in portable dental units (PDUs) for mobile dental treatments remains unclear. In this study, we aimed to investigate the factors affecting the effectiveness of PDU flushing.

**Materials and methods:**

Free residual chlorine concentration (FRCC) and heterotrophic plate counts (HPCs) in handpiece discharge water were evaluated as water quality indicators to determine the effectiveness of flushing PDUs, considering flushing duration, periods of PDU use, FRCC in the water used, pre-water drainage in water tanks and waterlines, and season. The effect of flushing on heterotrophic bacterial flora was analyzed by 16S rRNA gene sequencing.

**Results:**

PDUs with longer usage periods required a longer flushing duration for FRCC recovery. FRCC reached the standard level for water quality in Japan after at least 20 s of flushing. Flushing changed heterotrophic bacterial flora close to that of fresh tap water by reducing *Sphingomonas*. Flushing with purified water or stagnant tap water with insufficient FRCC did not allow the HPCs to reach the target level. Without pre-water drainage of the water tanks and waterlines in the summer, the HPCs did not always reach the target level, even with flushing.

**Conclusion:**

Periods of PDU use, pre-water drainage, water temperature, and FRCC substantially affect the effectiveness of PDU flushing. Flushing for at least 20 s with water containing appropriate free residual chlorine effectively restores the water quality of PDUs.

## Introduction

With the aging of several Asian societies, particularly in Japan and Taiwan,^1^^,^^2^ the demand for portable dental units (PDUs) has recently increased; these PDUs offer onsite treatment for home care residents.^3^^,^^4^ PDUs can provide dental healthcare to individuals in remote areas with limited transportation access or a shortage of dental professionals.^5^ Moreover, PDUs can be employed to treat acute dental symptoms in individuals compelled to stay at home or isolated due to infectious diseases, such as coronavirus disease 2019.^6^^,^^7^

A PDU comprises a unit body, water supply tank, vacuum tank, suction device, and dental unit waterlines (DUWLs). Usually, the unit is equipped with a vacuum syringe, ultrasonic scaler, micromotor, and multi-syringe. Water serves the dual purpose of cooling the heat generated by dental instruments, which can be detrimental to teeth, and cleaning tooth surfaces during dental procedures. DUWL contamination poses infection risks, with the potential inhalation of pathogenic microorganisms, such as *Pseudomonas aeruginosa* and *Legionella pneumophila*, during dental treatment and potential uptake from oral wounds.^8^^,^^9^ Therefore, PDUs should be equipped with a drainage system for draining water in water tanks and waterlines (WTWLs) after their use,^10^ ensuring a clean environment during mobile dental treatments. However, the available evidence does not conclusively indicate that the water drainage system in PDUs is adequate for effective infection control.

Assessing the number of heterotopic bacteria has been employed as a method to indicate the effectiveness of the water treatment process because they are predominant in tap water and can grow under low-temperature and low-nutrient conditions. Furthermore, they are associated with the disinfection process and increase in number with water retention and chlorine loss in the residual water of the water supply systems. Therefore, these bacteria serve as general indicators of water quality control.^11^ The Ministry of Health, Labour and Welfare (MHLW), Japan recommends that the heterotopic bacteria count of tap water should be 2000 CFU/mL or less.

Chlorine, a cost-effective disinfectant, has been widely employed in municipal water systems to inactivate microorganisms and impede their long-term development in the drinking water distribution system (DWDS).^12^ For instance, under the waterworks law in Japan, residual chlorine concentration in tap water should be above the minimum limit of 0.1 mg/L. Chlorine disinfects water by inhibiting essential cellular functions in microbes,^13^^,^^14^ for example, via the oxidation of cellular components, it inactivates or kills the bacteria. The total residual chlorine content is divided into combined residual chlorine and free residual chlorine (FRC). FRC can be used to inactivate pathogens and serve as an indicator of water potability.^15^ Maintaining a specific FRC concentration in tap water inhibits microorganism growth and prevents the formation of biofilms inside the DWDSs.^16^ However, information on the amount of FRC in PDUs remains unclear.

The Centers for Disease Control and Prevention (CDC), United States, recommends flushing waterlines of dental units for 20–30 s for each patient to improve water quality.^17^ However, flushing waterlines may remove only some of the accumulated suspended microorganisms and bacteria attached to the biofilm surface.^18^^,^^19^ Thus, the effect of flushing in PDUs with independent WTWLs that are structurally different from dental units in dental clinics is unknown.

Therefore, a flushing protocol that maintains water quality in PDUs for mobile dental treatments needs to be established. In this study, we aimed to investigate the factors (period of unit use, flushing duration, water temperature, chlorine concentration of the water used, and DUWL drains) that affect the effectiveness of PDU flushing.

## Materials and methods

### Experimental design and water collection

**Figure 1:**
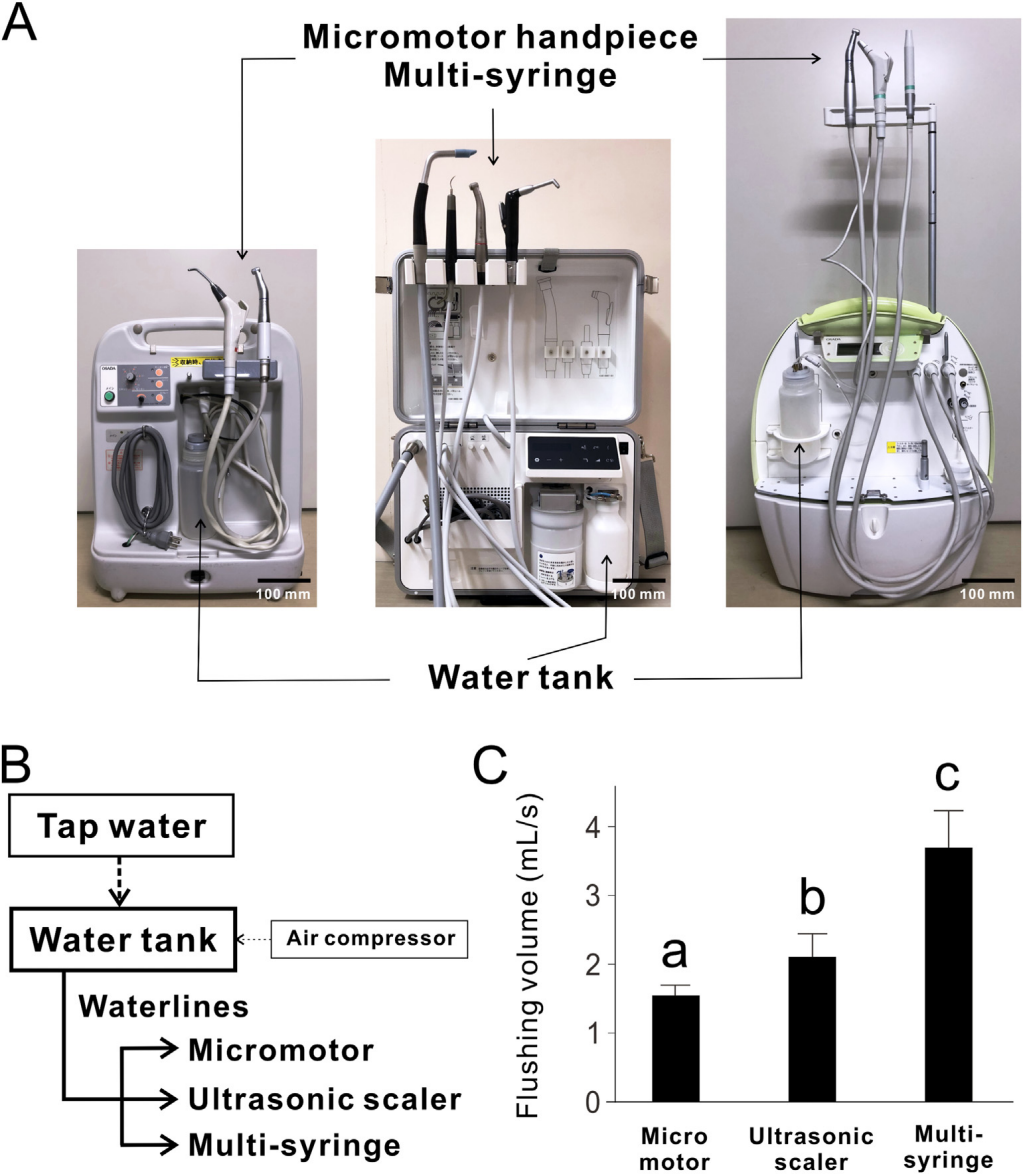
**Structure of water supply system for portable dental units (PDUs).** (A) Images of water tanks, handpieces, multi-syringes, and waterlines for PDUs. (B) Schematic diagram of the waterline for PDUs. (C) Flushing volume of waterline for each instrument. The data represent the mean ± standard deviation (n = 14–15). Different letters indicate statistically significant differences (*P* < 0.05, Steel–Dwass test).

In total, 15 different PDUs used in clinical practice or model practice (OPU-D2; Osada, Tokyo, Japan); (OPU-7G; Osada); (Portacube+; Morita Manufacturing, Kyoto, Japan) were investigated (Fig. 1A). All PDUs were maintained according to the following manufacturer's instructions. The PDUs used for less than 5 years and more than 10 years are defined as short- and long-PDUs, respectively. The water flow volume was set to maximum. For experiments to evaluate water quality in handpiece discharge of the PDUs, 15 mL of water discharged from the micromotor was sampled each time before and after flushing. The effect of the type of water loaded into the tank (running tap water, purified water, or stagnant tap water) was assessed (Fig. 1B). The running tap water was collected from the faucet after running water for 30 s. Commercial sterile deionized water (ASSWS-20; AS ONE Corp, Osaka, Japan) was used as the purified water. The stagnant tap water used in this study was the tap water collected first in the morning, which generally contains less chlorine because the water had been stagnant in the water supply pipes overnight. Water quality of stagnant water in PDUs in different seasons was also assessed. In this assessment, tap water was pooled for 1 week in the WTWLs of PDUs without water drainage both in summer and winter. The flushing ability of each water supplier in the PDUs was evaluated by measuring the volumes of water discharged from the handpieces of the micromotor, ultrasonic scaler, and multi-syringe.

### Measurement of free residual chlorine concentration

The free residual chlorine concentration (FRCC) was determined using the *N*, *N*-diethyl-*p*-phenylenediamine colorimetric assay with a residual chlorine analyzer (Photometer CL; Oyalox Co., Ltd., Tokyo, Japan).^20^ Ten mL of each sample was allowed to react with the reagents (DPD Plus; Oyalox Co., Ltd.) for 30 s. The FRCC measurements were performed within 2 h of water sampling at room temperature. For the FRCC, water quality was evaluated based on the tap water quality standard in Japan (≧0.1 mg/L) for each water sample.

### Bacterial plate count

Colony-forming unit (CFU) counts of heterotrophic bacteria were determined by counting colonies after aerobic incubation of 100 μL water samples in R2A agar (Nissui; Shimadzu Diagnostics Corp, Tokyo, Japan) at 20 °C for 7 days. The water quality for heterotrophic plate counts (HPCs) was determined based on the target value (≦2000 [3.30 log_10_] CFU/mL) of the Japanese tap water quality control.

Each 100 μL water sample was incubated aerobically on 5 % sheep blood agar (BD BBL™ CDC 5 % sheep blood agar medium for anaerobes; Becton Dickinson Japan, Tokyo, Japan) at 37 °C for 7 days to determine the nutrient-demanding bacteria species and their hemolytic potential.

### Bacterial community analysis with 16S rRNA gene sequencing

DNA from each experimental group was extracted from 96 different colonies isolated on R2A agar plates. The bacteria collected from the colony were added to 6 % w/v Chelex resin (InstaGene™ Matrix; Bio-Rad Laboratories, Inc., Hercules, CA, USA) and reacted. The supernatant was collected after being centrifuged. The extracted DNA was amplified by polymerase chain reaction (PCR) using the 16S rRNA gene-targeting primers 27F (5′-GCG TAT GCA ACT TGC CTT AC-3′) and 1482R (5′-GTT TCA ACG GCA GGC TGA AC -3′). The PCR test was performed using the HotStarTaq PLUS Master Kit (QIAGEN GmbH, Hilden, Germany). DNA sequencing was performed via the Sanger sequencing method with the thermal cycler and DNA analyzer (3730xl DNA Analyzer; Applied Biosystems [Thermo Fisher], Carlsbad, CA, USA) using the BigDye™ Terminator v3.1 Cycle Sequencing Kit (Applied Biosystems).

The consensus sequence of each sample was classified in the GenBank database using a web-based basic local alignment search tool (BLAST) optimized for highly similar sequences (MegaBLAST). The classified genus with the highest maximum score was regarded as the identity of the samples, excluding uncultured sample sequences and percent identity below 96 %.

### Statistical analysis

The flush volume for each handpiece was determined using the Kruskal–Wallis test, followed by the Steel–Dwass method as a multiple comparison method (*P* < 0.05). A two-way analysis of variance (ANOVA) examined the main effects and interactions between FRCC and HPCs (*P* < 0.05). When there was an interaction, a Tukey–Kramer honestly significant difference (HSD) multiple comparison test was performed (*P* < 0.05). A one-way ANOVA was used for stagnant and running tap water evaluation, followed by Tukey–Kramer HSD post-hoc tests (*P* < 0.05). Spearman's correlation coefficient was used to determine the correlation between the FRCC and HPCs. An analysis of covariance (ANCOVA) was used to correct for the influence of the variability of covariates on the main variates by season. All statistical analyses were performed using the JMP Pro software package, Version 17.1.0 (JMP Statistical Discovery LLC, Cary, NC, USA).

## Results

### Influences of flushing duration on water quality in PDUs

In all the units measured, the discharge flow rate of the micromotor handpiece was the lowest compared to those of the ultrasonic scaler handpieces and the multi-syringes (Kruskal–Wallis test corrected for multiple comparisons by the Steel–Dwass test; *P* < 0.05) (Fig. 1C).

**Figure 2:**
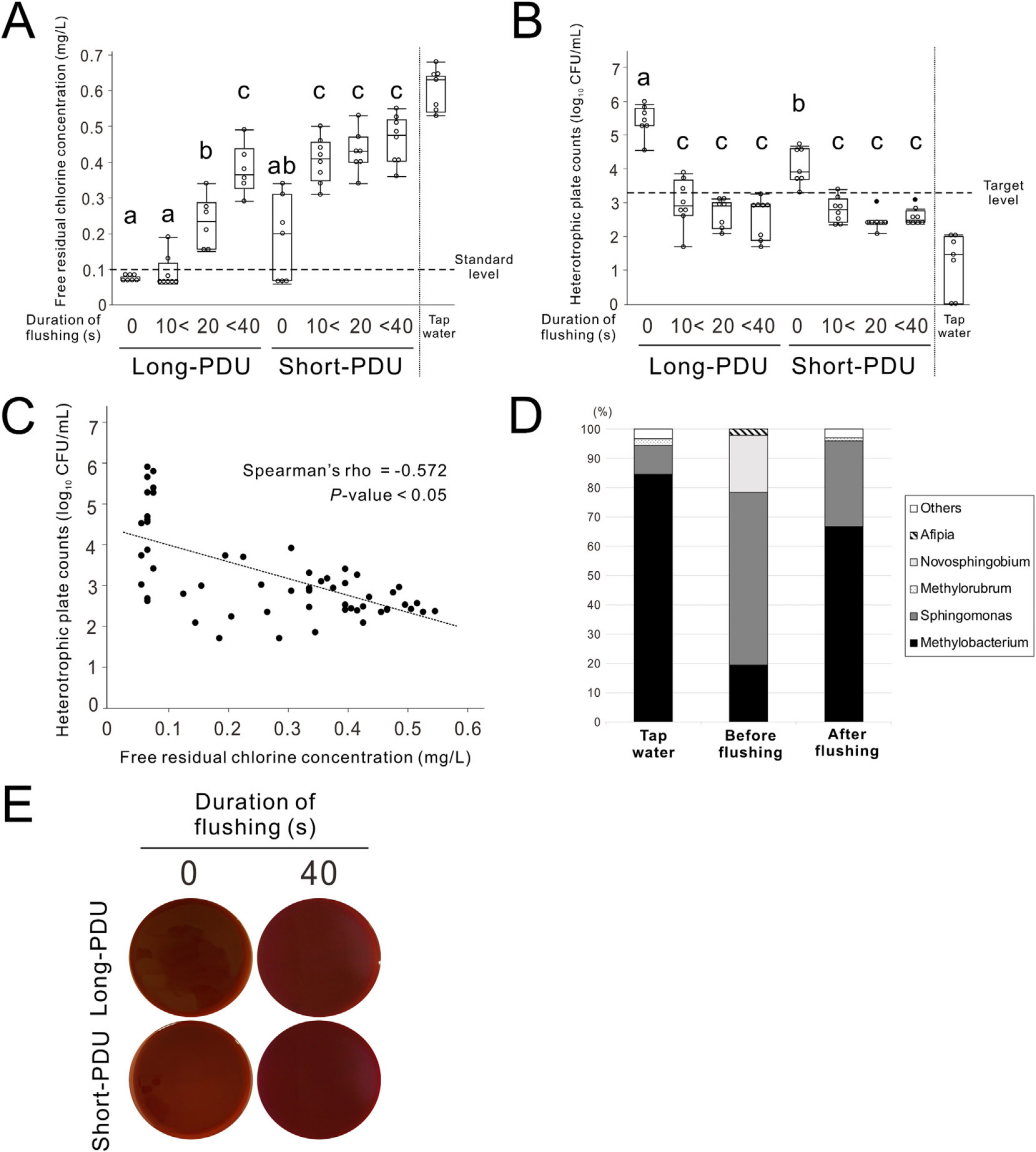
**Effect of flush duration on water quality in portable dental units (PDUs).** (A) Recovery of free residual chlorine concentration by flush duration per period of use for PDUs. (B) Reduction of heterotrophic plate counts through flush duration per period of use for PDUs. Data are presented as box plots with dot plots. (C) Correlation between heterotrophic plate counts and free residual chlorine concentration in the waterline for PDUs (n = 60, Spearman's rank correlation coefficient [R_s_]: −0.572). (D) Variation in the relative abundance of major operational taxonomic units at the genus level of detected heterotrophic bacteria. (E) Images of blood agar plates by flush duration in usage periods for PDUs. Different letters in (A) and (B) indicate statistically significant differences between them (*P* < 0.05, Tukey–Kramer HSD test; n = 6–8). CFU, colony forming units; Long-PDU, PDU used for more than 10 years; Short-PDU, PDU used for less than 5 years.

The average room temperature during the experiments was 23.3 °C, and the average water temperature of the handpiece discharge water from PDUs was 23.0 °C. There was a significant interaction in FRCC and HPCs in handpiece discharge water of PDUs between the flushing duration and usage period of PDUs, with a significant main effect for both (two-way ANOVA, *P* < 0.05) (Fig. 2A and B). The FRCC reached the standard level (≧0.1 mg/L: MHLW, Japan) for long-PDUs with over 20 s of flushing and for short-PDUs after less than 10 s (Fig. 2A). HPCs decreased to the target level (≦3.30 log_10_ CFU/mL: MHLW, Japan) with flushing of over 20 s, regardless of PDU usage period (Fig. 2B). A moderate negative correlation was observed between FRCC and HPCs in the PDUs (Spearman's rank correlation coefficient: −0.572, *P* < 0.05) (Fig. 2C).

Four to six bacterial genera were detected in running tap water or handpiece discharge water of long-PDU before and after flushing (Fig. 2D). Heterotrophic bacterial flora were characterized by *Sphingomonas* in pre-flushing handpiece discharge water and *Methylobacterium* in fresh tap water and post-flushing discharge water. No colonies were observed in any of the samples on blood agar plates after 7 days (Fig. 2E).

### Effects of purified water used for flushing on heterotrophic bacteria in the PDU waterline

**Figure 3:**
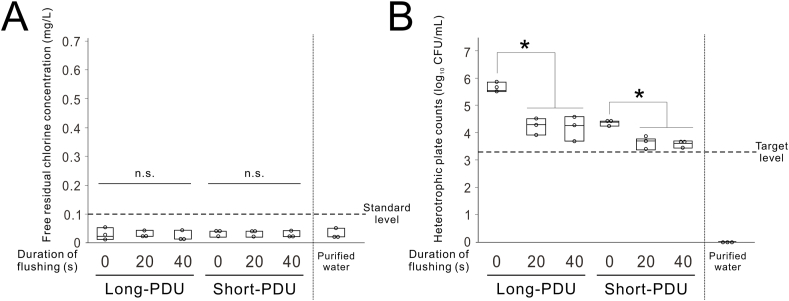
**Effect of purified water used for flushing on water quality in portable dental units (PDUs).** (A) Free residual chlorine concentration by flushing duration using purified water per period of use for PDUs. (B) Heterotrophic plate counts by flushing duration using purified water per period of use for PDUs. Data are presented as box plots with dot plots. ∗Statistically significant differences between them (*P* < 0.05, Tukey–Kramer HSD test; n = 3). n.s., non-significant difference. CFU, colony forming units; Long-PDU, PDU used for more than 10 years; Short-PDU, PDU used for less than 5 years.

The average room temperature during the experiments was 24.3 °C, and the average water temperature of the handpiece discharge water from PDUs was 23.5 °C. There was no significant interaction in both FRCC and HPCs in handpiece discharge water of PDUs between the flushing duration and PDU usage period (two-way ANOVA, *P* > 0.99, *P* = 0.069) (Fig. 3A and B). FRCC was hardly detected regardless of the flushing duration with purified water and PDU usage period (Fig. 3A). HPCs were slightly reduced by increasing the flushing durations but did not meet the target level (Fig. 3B).

### Effects of supplied tap water on heterotrophic bacteria in the PDU waterline

**Figure 4:**
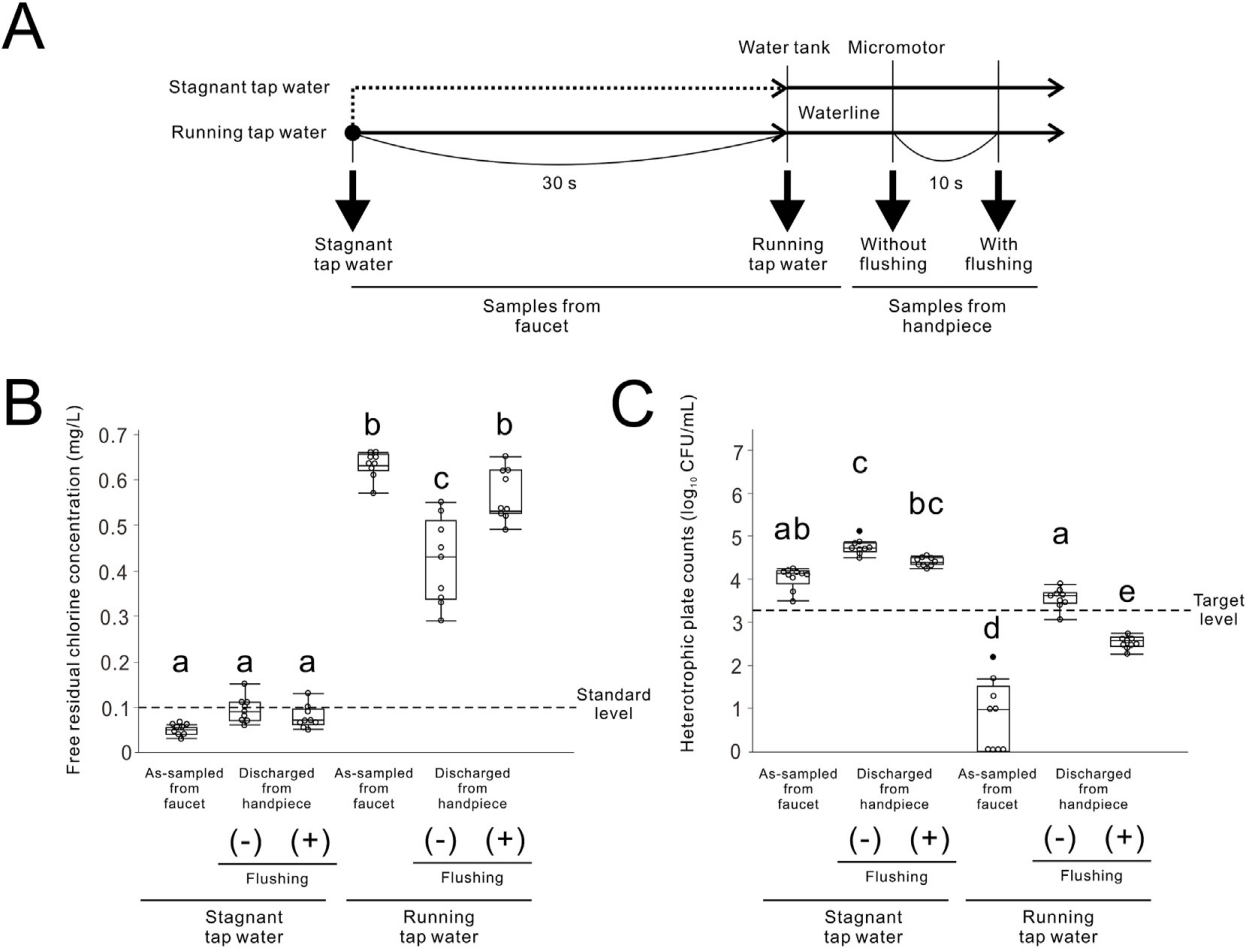
**Effect of supplied tap water on the water quality of portable dental units (PDUs).** (A) Experimental timeline for water sample collection. Stagnant tap water is the tap water collected first in the morning from the faucet, which has been stagnant in the water supply pipes overnight. (B) Free residual chlorine concentration in PDUs with and without a pre-flush of supplied tap water. (C) Heterotrophic plate counts in PDUs with and without a pre-flush of supplied tap water. Data are presented as box plots with dot plots. Different letters in (B) and (C) indicate statistically significant differences between them (*P* < 0.05, Tukey–Kramer HSD test; n = 9). CFU, colony forming units.

To evaluate the effect of FRC in tap water on bacterial reduction in the DUWL of the PDUs by flushing, stagnant or running tap water was loaded into water-supply tanks. FRCC and HPCs were measured at each flushing duration point (Fig. 4A). The average room temperature during the experiments was 19.2 °C, and the average temperatures of the water discharged from the handpiece from the PDUs loaded with stagnant and running tap water were 24.1 °C and 18.6 °C, respectively. Stagnant tap water was inherently low and high in the FRCC and HPCs, respectively (Fig. 4B and C). Flushing with stagnant tap water did not improve those values in the handpiece discharge water of the PDUs.

### Effects of pre-water drainage in WTWLs and seasons on water quality in PDUs

**Figure 5:**
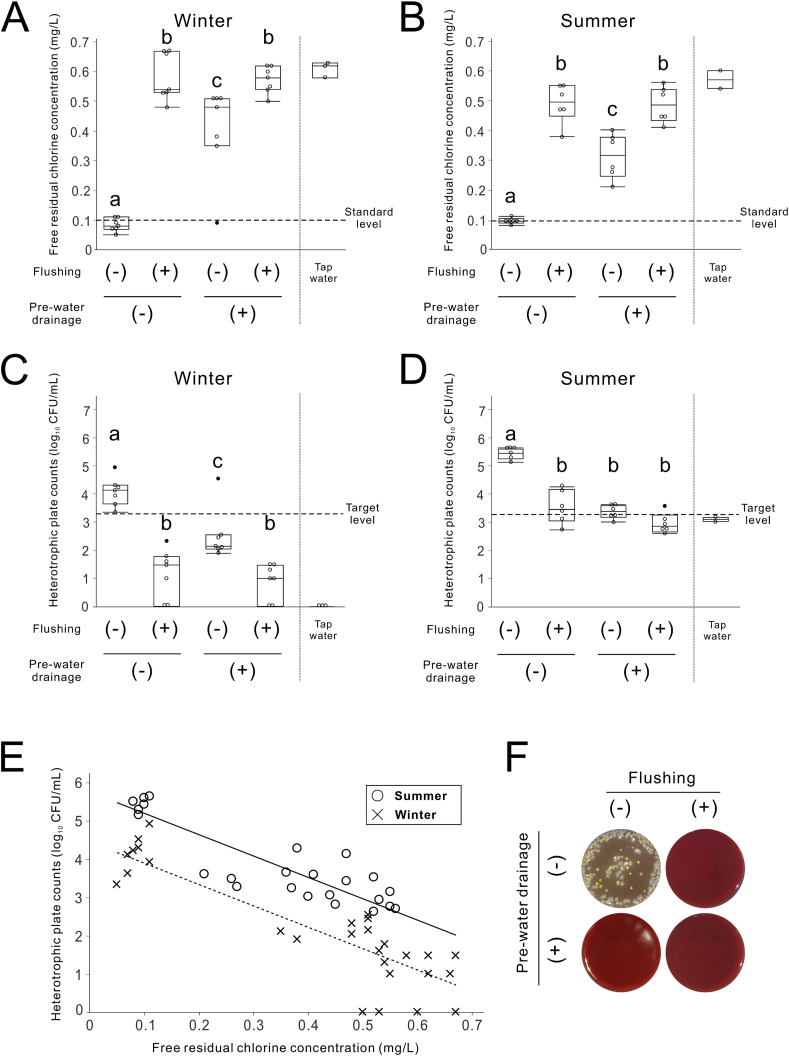
**Effects of pre-water drainage in water tanks and waterlines (WTWLs) and seasons on water quality in portable dental units (PDUs).** (A, B) Recovery of free residual chlorine concentration by draining the WTWLs and flushing the waterlines for PDUs in winter and summer, respectively. (C, D) Reduction of heterotrophic plate counts by draining the WTWLs and flushing the waterlines for PDUs in winter and summer, respectively. Data are presented as box plots with dot plots. (E) Correlation between heterotrophic plate counts and free residual chlorine concentration in the waterline for PDUs (analysis of co-variance using free residual chlorine concentration as a covariate; n = 52). (F) Images of blood agar plates from PDUs with and without water tank drains and flushing during the summer. Different letters in (A–D) indicate statistically significant differences between them (*P* < 0.05, Tukey–Kramer HSD test; n = 6–7). CFU, colony forming units; Pre-water drainage, drainage in WTWLs.

The average room temperatures during the experiments in winter and summer were 7.0 °C and 26.5 °C, respectively. The average water temperatures of the handpiece discharge water from PDUs in winter and summer were 11.2 °C and 24.6 °C, respectively. Regardless of the season, under the conditions of no draining and no flushing, FRCC and HPCs in the handpiece discharge water of the PDUs were markedly low and high, respectively (Fig. 5A–D). There was a significant interaction in both FRCC and HPCs in handpiece discharge water of PDUs between the flushing and pre-water drainage in WTWLs, with a significant main effect for both (two-way ANOVA, *P* < 0.05) (Fig. 5A–D). Flushing significantly improved FRCC even under conditions without pre-water drainage in both winter and summer (Fig. 5A and B). HPCs in the PDUs without pre-water drainage were also significantly reduced by flushing, but the decrease amount was apparently low in the summer and did not adapt to the target level (Fig. 5C and D).

There was no significant interaction between the FRCC and season in the ANCOVA test using the FRCC as a covariate (*P* = 0.510). HPCs were significantly higher in summer than in winter after correcting for FRCC using the ANCOVA (Student's t-test, *P* < 0.05) (Fig. 5E). Except for the samples from PDUs without pre-water drainage or flushing, no colonies were detected on blood agar plates culturing handpiece discharge water from PDUs. Handpiece discharge water from the PDUs without pre-water drainage or flushing grew white and yellow colonies with hemolytic activity (Fig. 5F).

## Discussion

While DUWL contamination in fixed-type dental units has been discussed, there is limited knowledge regarding contamination of PDU waterlines and water quality control. To our knowledge, this is the first study to suggest the effectiveness of flushing for managing water quality in PDUs.

Although there are no legally binding regulations on water quality in dental units in Japan, MHLW recommends that the number of heterotrophic bacteria should not exceed 2000 CFU/mL (3.30 log_10_ CFU/mL), the control target level for drinking water. CDC guidelines for infection control in the dental environment recommend that HPCs in dental unit water should not exceed 500 CFU/mL (2.70 log_10_ CFU/mL).^17^ This value was set based on the drinking water standards established by the U. S. Environmental Protection Agency. HPC is measured by incubation at 35 °C for 48 h using the pour plate method with R2A agar.^21^^,^^22^ In Europe, one study evaluated the water quality of DUWL based on the drinking water standards at that time (aerobic colony count of less than 100 CFU/mL [2.00 log_10_ CFU/mL] after incubation at 22 °C for 72 h using the pour plate method with yeast extract agar).^23^^,^^24^ Direct comparisons of our results and previous findings regarding the HPCs might be difficult because of the different media, incubation temperatures, and incubation periods.

The results showed that PDUs with longer periods of use tended to require more flushing duration to reduce HPCs (Fig. 2B). Our findings are consistent with previous reports that years of use of fixed-type dental units correlate with HPCs and affect the rate of decrease of bacteria in flushing.^25^^,^^26^ It seems that a mature and robust biofilm is formed in DUWLs that have been in use for a longer period.

The results suggest that eliminating microbial contamination in PDU waterlines by flushing with purified water alone is difficult (Fig. 3A and B), which is consistent with previous reports that flushing waterlines of fixed-type dental units with distilled water alone does not reduce bacterial counts to the recommended levels.^27^ Therefore, maintaining proper FRC in tap water is important to maintain low bacterial counts. To minimize changes in water quality during distribution and reduce bacteria growth during transport, different countries use different concentrations of chemical substances (including free chlorine, chlorine dioxide, and monochloramine) to maintain disinfectant residues in water.^28^, ^29^, ^30^ However, to prevent toxic effects and excessive by-products from chlorine disinfection, the World Health Organization recommends that free chlorine in drinking water should not exceed 5 mg/L.^31^ Natural organic matter in the raw water reacts with chlorine and forms disinfection by-products such as trihalomethanes, haloacetic acids, and halogenated acetonitrile. These compounds degrade water quality and have been epidemiologically confirmed to be closely associated with malignant organ growth.^15^ Therefore, maintaining an optimal FRCC is critical.

The number of bacteria in the handpiece discharge water from the DUWLs of the PDU increased during the summer months when the water temperature was higher (Fig. 5E). Our results corroborate previous studies that show increased bacterial counts in tap water during the summer months.^30^^,^^32^ Water temperature is an important factor that affects bacterial growth rates.^33^ High temperature is also suggested to promote bacterial attachment and biofilm formation in DWDS.^34^ Furthermore, FRC is negatively correlated with tap water temperature and is attenuated during summer.^35^^,^^36^ Therefore, water quality monitoring of PDUs in areas with high temperatures and during the summer should be strictly controlled.

The *Methylobacterium* genus detected in this study is ubiquitous in the environment, occurring in the air, soil, plants, and freshwater (Fig. 2D). It is referred to as “pink-pigmented facultative methylotrophs” because it forms pink-colored colonies.^37^ Although less pathogenic, it can cause infections in immunocompromised patients.^38^ Despite the correlation between FRCC and HPC, a certain number of bacteria remain even when FRCC increases (Figure 2, Figure 5E). The remaining bacteria could be chlorine-resistant *Methylobacterium*.^39^

This study has some limitations. First, the protocol for an adequately maintained PDU may restrict its application to contaminated PDUs. Second, the heterotrophic bacteria count does not comprehensively reflect the maturity of the formed biofilm. Third, the absence of seasonal variations may limit the generalizability of findings to specific environmental conditions. Fourth, tap water quality varies by country and region, and the study's focus on Japanese regulations may restrict the applicability of the proposed water safety management protocol to other regions. Future research should address these limitations to enhance the robustness and generalizability of our findings.

In conclusion, clinically acceptable water quality in PDU waterlines requires flushing with clean water containing a sufficiently high FRCC for at least 20 s. Drainage of WTWLs is also required to prevent bacteria growth, particularly at high room temperature. This study may help to establish a water safety management protocol for PDUs. Future development of water quality monitoring and management systems for PDUs is important.

## Declaration of competing interest

The authors have no conflicts of interest relevant to this article.
